# Conductive Polyaniline Doped with Dodecyl Benzene Sulfonic Acid: Synthesis, Characterization, and Antistatic Application

**DOI:** 10.3390/polym12122970

**Published:** 2020-12-12

**Authors:** Cheng-Ho Chen, Jing-Mei Wang, Wei-Yu Chen

**Affiliations:** Department of Chemical and Materials Engineering, Southern Taiwan University of Science and Technology, Tainan City 710, Taiwan; ma340104@stust.edu.tw

**Keywords:** polyaniline, dodecyl benzene sulfonic acid, xylene, chemical oxidative polymerization, conductive composite film

## Abstract

A novel method was conducted to synthesize conductive polyaniline (PANI) doped with dodecyl benzene sulfonic acid (DBSA) (PANDB) in xylene by using chemical oxidative polymerization at 25 °C. Meanwhile, the synthesis process was photographed. Results showed as the reaction time was increased, and the color of the product was gradually turned into dark green. The influence of different synthesis time on properties of synthesized PANDB was then examined by a Fourier transform infrared (FTIR) spectrometer, an ultraviolet-visible spectrophotometer (UV-vis), a four-point measurement method, and a Field-emittance scanning electron microscope (FE-SEM). The result indicated that the optimum reaction time was 24 h with conductivity at around 2.03 S/cm. FE-SEM images and the conductivity testing showed that the more needle-like shapes in resulted PANDB, the higher the conductivity. The synthesized PANDB solution was blended with UV curable coating firstly and then coated on polyethylene terephthalate (PET) sheet. The UV coating/PANDB conductive composite films displayed an impressive translucency along with an adequate flexibility at room temperature. The UV coating/PANDB conductive composite film on PET sheet was flexible, transparent, and with antistatic function.

## 1. Introduction

Inherently conductive polymers (ICPs) are a specific category of synthetic polymers with distinctive electro-optic properties [1]. These polymers have conjugated chains with alternating single and double bonds. The π-electrons in the ICPs, which are highly delocalized and simply polarizable, show an imperative role in the electro-optic properties of ICPs. The first conductive polymer, iodine-doped polyacetylene, was discovered by Alan Heeger, Alan MacDiarmid, and Hideki Shirakawai in 1977. They were awarded the Nobel Prize in chemistry for this discovery in 2000. Polyacetylene (PAs), polypyrrole (PPy), polythiophene (PTh), polyfuran (PFu), and polyaniline (PANI) were examples of ICPs [2]. Among ICPs, PANI has received considerable attention in various industrial and biomedical fields due to their facile preparation, low cost, tunable conductivity, biocompatibility, low toxicity, attractive electro-optic properties, and environmental stability [3]. Synthetic materials based on PANI are commonly used in high-tech sectors, such as marine antifouling, plastic metal anticorrosion technology, electromagnetic shielding, stealth technology, antistatic technology and sensor components, electroluminescent, solar battery, secondary battery materials, catalytic materials, and anti-corrosion materials [4].

However, if the reaction time was not long enough, the PANI synthesis process might produce toxic by-products (benzidine and trans-azobenzene) [5]. Meanwhile, PANI has also some processing disadvantages, such as low solubility or insolubility in the most common solvents, infusibility, and weak processability [1]. Many attempts have been made to synthesize soluble and conductive PANI doped with various dopants. The most attractive and promising system was synthesis of PANI doped with dodecyl benzene sulfonic acid (DBSA) (PANDB) in aqueous solution [6,7,8] or in emulsion polymerization [9,10]. In these synthesis systems, DBSA can act both as surfactant and dopant in the synthesis process. As-synthesized PANDB then became soluble in organic solvents. This was because the molecular chain of DBSA has lipophilic group (–C_12_H_25_). It was favorable to dissolve PANDB in common organic solvents. Thus, the PANDB-based materials have appropriate applications such as antistatic materials [9,10,11,12,13], electrostatic discharge, electromagnetic interference (EMI) shielding materials, anticorrosion coatings [14,15,16], batteries [17], sensors [18], etc.

Wang et al. prepared the conductive PANDB/PI microfiber membranes by polymerized PANDB on the surface of PI nanofiber membrane. The PANI/PI microfiber membranes had potential use in novel functional fiber and fabric applications, including antistatic and electromagnetic shielding fabric, electrical resistive heating fabric and intellectual fabric [11]. Chu et al. reported that the conductive PANDB was synthesized from aniline salt through in situ polymerization in a well-dispersed aqueous solution of water-based polyurethane (WPU). Additionally, the composite coatings with excellent conductivity have offered a pathway for the different applications in antistatic, electrostatic discharge (ESD), and electromagnetic interference (EMI) shielding materials [12].

However, the PANDB synthesized in aqueous solution or in emulsion polymerization could not be properly dissolved in organic solvents. Thus, in order to improve the solubility of PANDB in xylene, a novel polymerization method was developed to synthesize PANDB in xylene directly in this study. The influence of synthesis time on properties of synthesized PANDB was examined by a Fourier transform infrared (FTIR) spectrometer, an ultraviolet-visible spectrophotometer (UV-vis), a four-point measurement method, and a Field-emittance scanning electron microscope (FE-SEM). The synthesized PANDB was blended with UV curable coating firstly and then coated on polyethylene terephthalate (PET) sheet. The translucency and antistatic function of UV coating/PANDB conductive composite film were also examined.

## 2. Experimental

### 2.1. Materials

Aniline (AN) and ammonium persulfate (APS) were purchased from Merck (Darmstadt, Germany). Benzoyl peroxide (BPO) and xylene were purchased from Shimakyu Chemical Co. (Osaka, Japan). Dodecylbenzene sulfonic acid (DBSA) was purchased from Tokyo Kasei (Tokyo, Japan). UV curable coatings were provided by Eternal Materials Co., Ltd. (Kaohsiung, Taiwan).

### 2.2. Synthesis of Emeraldine Salt (ES) Type PANI Doped with DBSA (PANDB)

PANDB synthesized in xylene was illustrated as below. Firstly, 6.2 g of BPO (0.026 mol) was added into a beaker containing 100 mL of xylene under constant stirring until completely dissolved (solution A). Then, 3.0 g of AN (0.032 mol) and 8.4 g of DBSA (0.026 mol) were added in another beaker containing 100 mL of xylene under constant stirring until completely dissolved (solution B). After that, pouring the solution A into the solution B for chemical oxidative polymerization of AN at 25 °C. The reaction time was controlled at 6 h, 12 h, 24 h, 36 h, and 48 h, respectively. When the reaction time was reached, reverse osmosis (RO) water was poured into the reaction solution and then the excess DBSA was filtered out. The resulted product was dark green emeraldine salt (ES) type conductive PANI doped with DBSA (PANDB) and it could be well dispersed in xylene. In order to obtain dried PANDB for characterization, the product solution was dried in an oven at 80 °C.

In order to compare the dispersibility and solubility of PANDB synthesized in xylene and in aqueous solution, we followed the previous experimental procedure to synthesize PANDB in aqueous solution [19]. In that study, APS was the initiator used for the oxidative polymerization of AN in aqueous solution.

### 2.3. Preparation UV Coating/PANDB Conductive Composite Film

The synthesized PANDB (5 wt% and 10 wt%) with the optimized conductivity was added into a fixed proportion of UV curable coating. The UV coating/PANDB conductive composite solution was subjected to ultrasonic treatment for 10 min and then a spin coater (model: spin coater kw-4a, Chemat Technology Co., Ltd., Los Angeles, CA, USA) was used to coat UV curable coating, UV coating/PANDB conductive composite solution on a flexible and translucency PET sheet. At room temperature, 1 mL UV curable coating, UV coating/5 wt% PANDB, and UV coating/10 wt% PANDB conductive composite solutions were dropped on the surface of the PET sheet, respectively. Then, the rotation speed of the spin coater was set at two stages. The first stage was set at 500 rpm for 5 s. The second stage was set at 1200 rpm for 10 s. The coated UV coating and UV coating/PANDB conductive composite films were put into an oven for drying at 80 °C for 10 min in order to volatilize the solvent. After that, the dried UV coating and UV coating/PANDB conductive composite film were irradiated with a wavelength of 365 nm UV lamp for 30 min to complete the curing process of UV curable coating.

### 2.4. FTIR Analysis

The chemical structures of samples synthesized in reactions with differing durations were identified using a Fourier transform infrared (FTIR) spectrometer (Spectrum One; Perkin Elmer, Waltham, MA, USA). The samples and dehydrated potassium bromide (KBr) (weight ratio of sample/KBr = 1/99) were grounded together into fine powders and then the homogeneous mixtures were pressed to form pellets for analysis. The wavenumber was ranged from 600–4000 cm^−1^, and the scanning rate was 64/s.

### 2.5. UV-Vis Analysis

The samples were well dispersed in xylene at room temperature. Then, optical absorbance of the PANDB solution in the wavelength range of 250–900 nm was determined using an ultraviolet-visible spectrophotometer (UV-vis; Shimadzu, model UV-2401 PC, Kyoto, Japan). The UV–vis spectrophotometer was also used to examine the degree of transparence of the UV coating/PANDB conductive composite film coated on the surface of PET sheet.

### 2.6. DC Electrical Conductivity Test

0.1 g PANDB was weighed and then pressed under 3.0 × 10^5^ psi for 2 min at room temperature. The electrical conductivity σ (S/cm) of the sample was examined by a four-point probe meter (model: LSR4-KHT200, KeithLink Technology Co., Ltd., Taipei, Taiwan) at room temperature. Each synthesized PANDB was firstly compressed into a sample with a diameter of 10 mm. Then, 10 different locations on the sample surface were measured. The obtained results were averaged and reported. The surface resistance (Ω/sq.) of PET sheet, pure UV-coating film, and UV coating/PANDB conductive composite films were also examined and reported at room temperature by the same instrument.

### 2.7. Field-Emittance Scanning Electron Microscope (FE-SEM)

The surface morphology of PANDB synthesized at different times was investigated by a Field-emittance scanning electron microscope (FE-SEM) at 10 kV with a JEOL (Tokyo, Japan) JSM-840A scanning microscope. All specimens were coated with a conductive layer of sputtered platinum.

## 3. Results and Discussion

### 3.1. The Process of Synthesizing PANDB in Xylene

**Figure 1:**
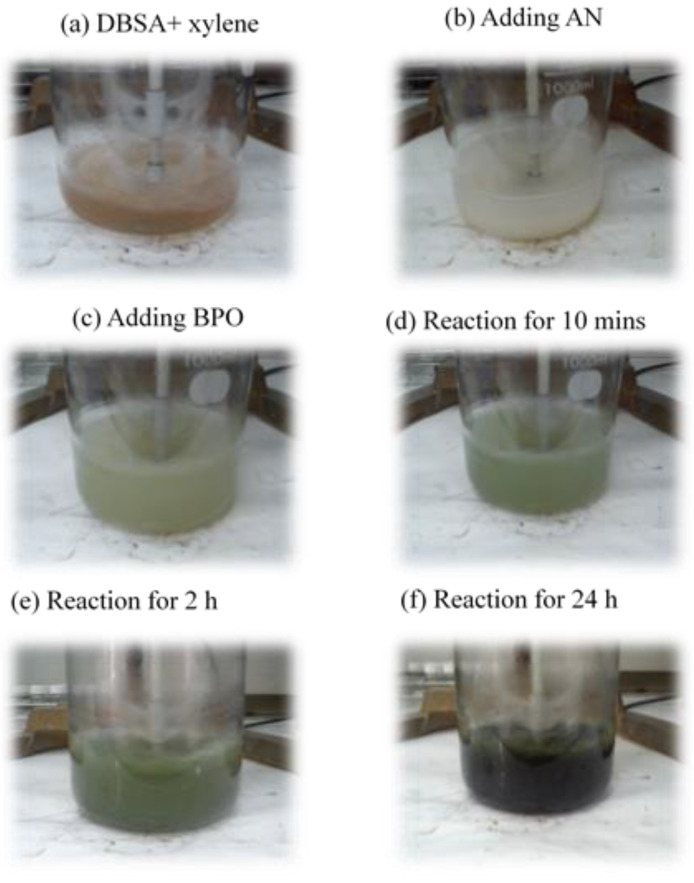
The synthesis process of PANDB with the molar ratio of AN:BPO:DBSA = 1.0:0.8:0.8 in xylene for 24 h at 25 °C.

The process of synthesized conductive PANDB with the molar ratio of AN:BPO:DBSA equal to 1.0:0.8:0.8 was shown in Figure 1. Figure 1a showed DBSA was dissolved in xylene to form a light orange solution. Then, AN was added into the previously described solution (Figure 1b), and a milky white complex was formed. When adding 100 mL BPO xylene solution into the mentioned complex, the color of mixture was changed as the reaction time was increased. Figure 1c–f showed the color of reaction mixture was slowly changed from light green to dark green. This was because the PANI doped with DBSA to form ES type conductive PANDB. This observation also indicated the formation of a polaron band [20].

**Figure 2:**
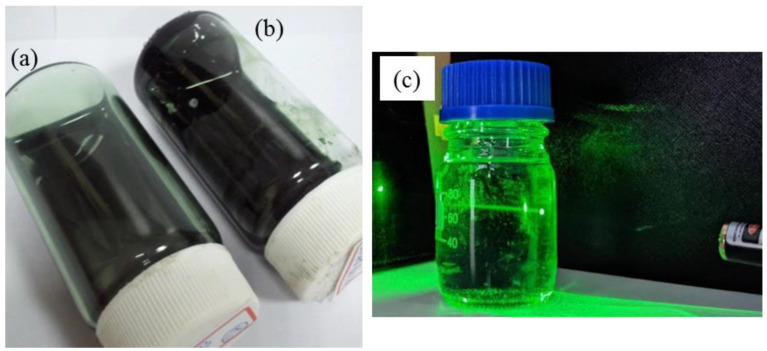
Photographs of PANDB dissolved in xylene: (**a**) PANDB synthesized in xylene; (**b**) PANDB synthesized in aqueous solution; and (**c**) PANDB synthesized in xylene showing the Tyndall effect.

If PANDB synthesized in xylene and in aqueous solution were respectively dissolved in xylene, it can be found that most of the PANDB synthesized in xylene can be uniformly nano-dispersed and a small part of PANDB can be even dissolved in xylene (no precipitation, see Figure 2a). However, PANDB synthesized in aqueous solution cannot be uniformly dispersed nor dissolved in xylene (with obvious precipitation, see Figure 2b). Meanwhile, the PANDB synthesized in xylene also showed the Tyndall effect (see Figure 2c). This was because the solubility issue of the adopted synthetic solvents, which resulted in the PANDB molecules in product to be arranged differently during the synthesis process. The solubility parameter of PANDB in water (23.4 (cal/cm^3^)^1/2^) was significantly higher than that in xylene (8.8 (cal/cm^3^)^1/2^). As a result, the PANDB synthesized in aqueous solution may be arranged tightly among the molecules. Therefore, PANDB synthesized in aqueous solution cannot be uniformly nano-dispersed and dissolved in xylene.

### 3.2. Characterization of PANDB Synthesized in Xylene

**Figure 3:**
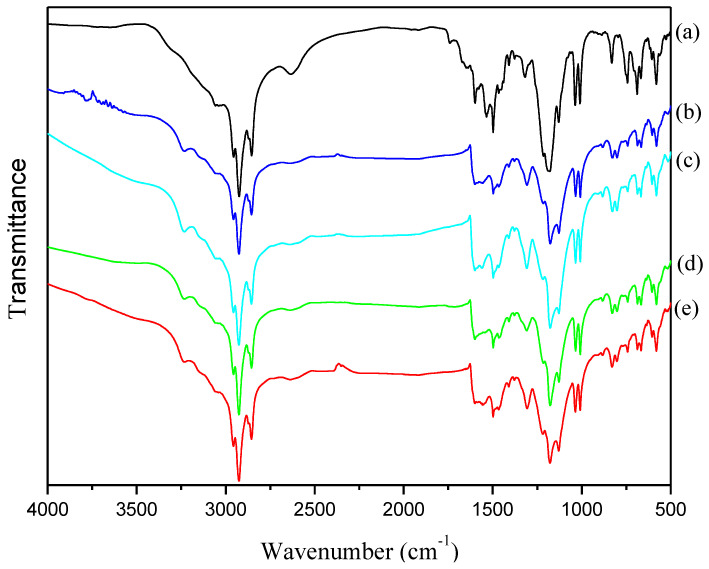
FT-IR spectra of PANDB synthesized in xylene at 25 °C: (**a**) 6 h; (**b**) 12 h; (**c**) 24 h; (**d**) 36 h; and (**e**) 48 h.

Figure 3 showed the FT-IR spectra of PANDB synthesized in xylene under different reaction times. There was no obvious difference from the FT-IR analysis results. The characteristic peaks appearing at around 2800~3000 cm^−1^ were due to the stretching vibration of –CH_3_ and –CH_2_ of PANDB. The characteristic peaks appearing at around 1590 and 1490 cm^−1^ were due to the stretching vibrations of quinoid ring (N***=***Q***=***N) and benzenoid ring (N–B–N), respectively. The characteristic peak appearing at around 1300 cm^−1^ was attributed to C–N stretching vibration of the secondary amine in the PANDB backbone. The characteristic peak appearing at around 1030 cm^−1^ was the absorption peak of the stretching vibration of the sulfonic acid group (-SO_3_H), which was part of the DBSA molecular structure. Additionally, the characteristic peak appearing at 829 cm^−1^ was due to aromatic C–H bending vibration band due to the 1, 4-disubstituted benzene ring.

**Figure 4:**
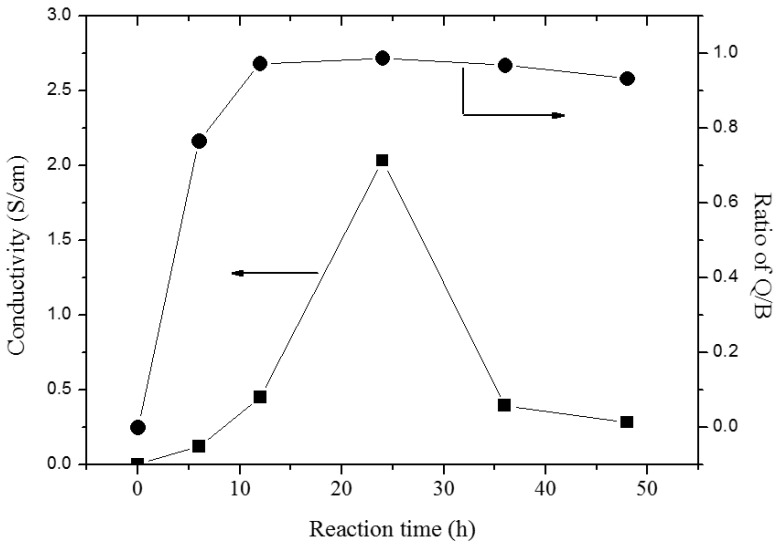
Relationship among reaction time, conductivity and the ratio of Q/B from FTIR (Fourier transform infrared) measurements. (Q/B: the ratio of area of peaks corresponding to quinoid (Q) and benzenoid (B) structure present in the PANDB).

From the ratio of peak areas corresponding to the 1590 and 1490 cm^−1^, the ratio of quinoid (Q) to benzenoid (B) structure present in the PANDB, which indicated the oxidation level of synthesized PANDB, can be estimated [21]. Figure 4 showed the relationship among reaction time, conductivity and the ratio of Q/B. For reaction time at 6 h, 12 h, 24 h, 36 h, and 48 h, the Q/B values were 0.765, 0.972, 0.987, 0.968, and 0.933, respectively. Meanwhile, the conductivity for reaction time at 6 h, 12 h, 24 h, 36 h, and 48 h, were 0.12, 0.45, 2.03, 0.39, and 0.28 S/cm, respectively. The conductivity was increased with increasing the reaction time. When the reaction time was 24 h, the conductivity is the highest. The higher Q/B value, the higher the conductivity. The results showed when the reaction time was 24 h, the synthesized PANDB reached the optimized Q/B and conductivity. The result illustrated that if the reaction time was too short, the chemical oxidation reaction was insufficient. Meanwhile, if the reaction time was too long, it might result in an excessive oxidation and other side reactions. Both phenomena could affect the conductivity of the product. Thus, in this synthesis system, the optimum reaction time was founded as 24 h.

**Figure 5:**
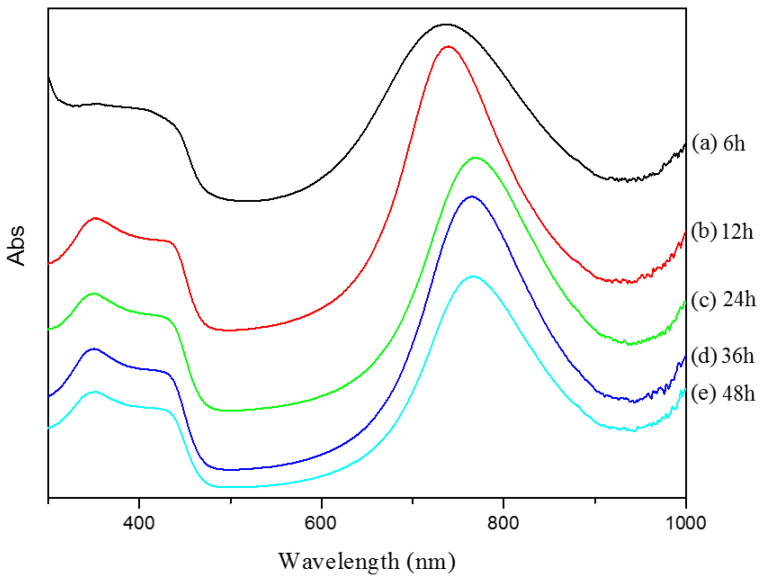
UV-vis absorption spectra of PANDB synthesized in xylene at different reaction times.

Figure 5 demonstrated the UV-vis absorption spectra of PANDB synthesized in xylene under different reaction times. All spectra showed two small bands; one at approximately 350 nm and the other at 450 nm. Meanwhile, all spectra were along with a larger, broader band around 730~800 nm with the characteristic “free carrier tail” extending past the UV-vis region (>900 nm) [22]. The absorption peak appearing at around 350 nm was ascribed to *π*–π* transition of the benzenoid rings on the PANDB backbone. The peaks appearing at around 450 and 800 nm were attributed to polaron–π* transition and excition transition of quinoid rings, respectively. The second band at 450 nm was attributed to the polaron–π* transition, or quinoid rings. The broad band corresponds to the π–polaron transition, or formation of the exciton. The bands at 450 and 730~800 nm both involved formation of the polaron and were greatly affected by the level of doping of the polymer. These peaks were shown to be characteristic of doping with DBSA, although these peaks can shift slightly according to the degree of doping, conjugation length and solvent used to synthesize PANDB [23].

**Figure 6:**
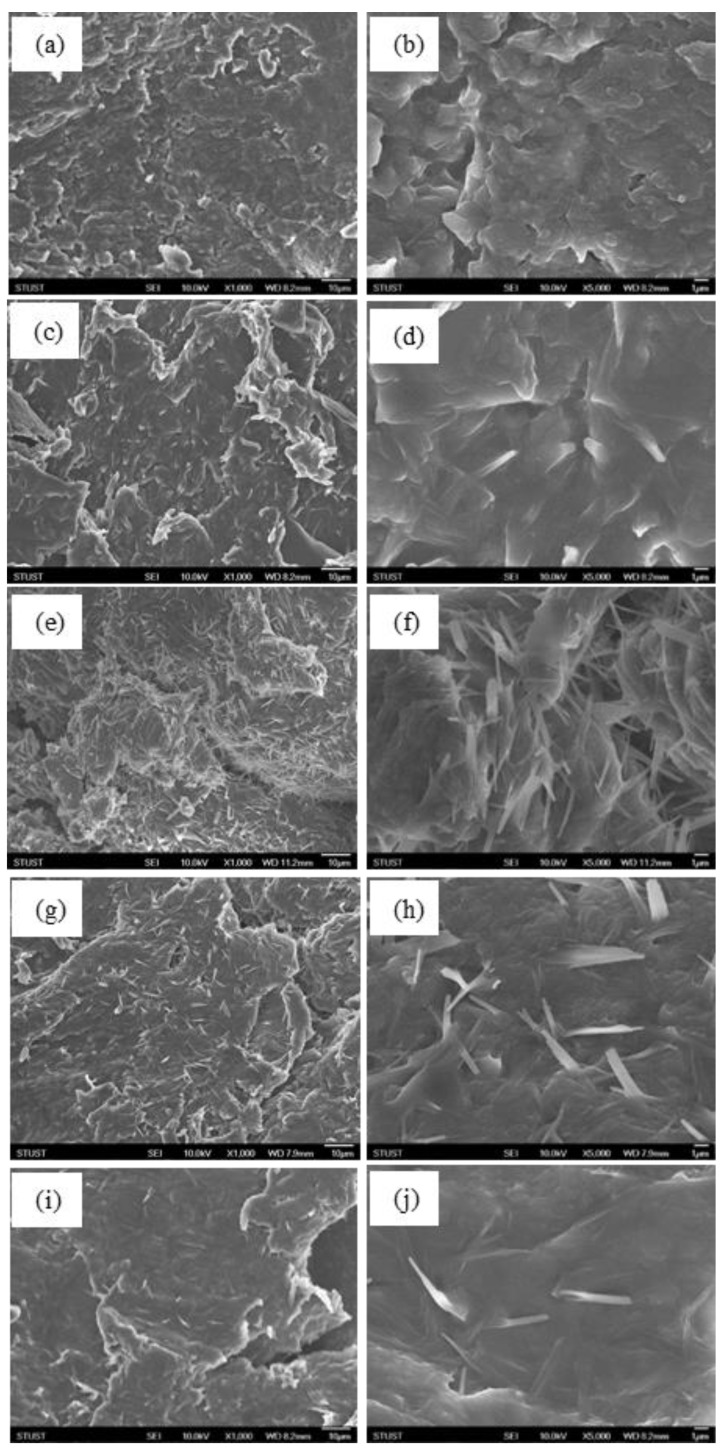
FE-SEM images of the fractured surfaces of PANDB synthesized in xylene at different reaction time: (**a**) 6 h; (**c**) 12 h; (**e**) 24 h; (**g**) 36 h; and (**i**) 48 h (×1000); (**b**) 6 h; (**d**) 12 h; (**f**) 24 h; (**h**) 36 h; and (**j**) 48 h (×5000).

Figure 6 showed FE-SEM images of the fractured surfaces of PANDB synthesized in xylene under different reaction times. The left images (Figure 6a,c,e,g,i) were taken under a low magnification (×1000) and the right ones (Figure 6b,d,f,h,j) were taken at a high magnification (×5000). Comparing the FE-SEM images at small magnification (×1000), the morphologies of fractured surfaces of PANDB synthesized at different reaction time were all similar. However, the amount of needle-like particles in the product was increased as the reaction time was increased. The optimized amount was observed when the reaction time was 24 h, as shown Figure 6e. Meanwhile, the same phenomenon was observed when the large magnification (×5000) was adopted, as shown in Figure 6f. Furthermore, in Figure 6a,b, the morphologies of fractured surfaces of PANDB synthesized at 6 h did not show an obvious needle-like shape. At the same time, it could be found when the reaction time was 36 h as shown in Figure 6h, and 48 h as shown in Figure 6j, the needle-like particles were tended to be decreased by comparing with those at 24 h.

Appropriate reaction time resulted in the increasing of needle-like particles in products and also increasing the inner contact surface area of products, which might be the reason for the higher conductivity. Therefore, the optimum reaction time was set as 24 h in this synthesis system. According to our previous research results, different synthesis conditions can result in different surface morphology of the product [19]. This was a very interesting phenomenon, and it was worth continuing to explore and study.

**Figure 7:**
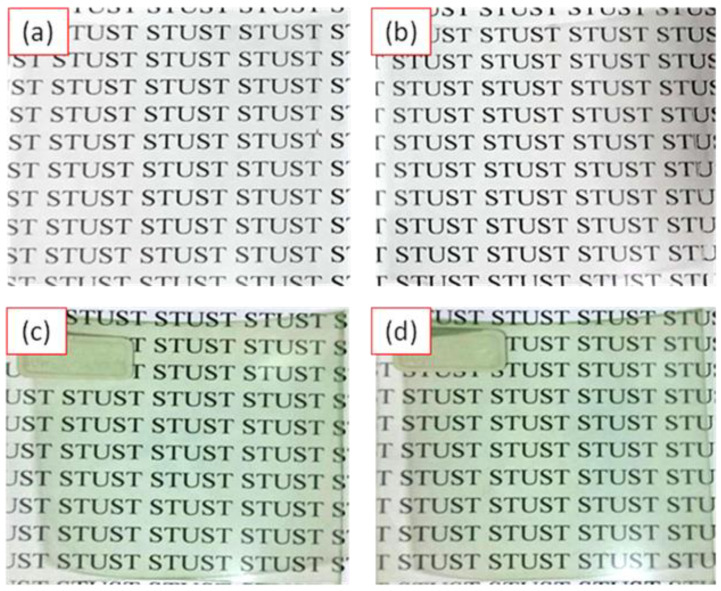
Photograph of (**a**) the PET sheet; (**b**) UV coating; and (**c**) UV coating/5 wt% PANDB conductive composite film; and (**d**) UV coating/10 wt% PANDB conductive composite film deposited on PET sheets. The abbreviation of the school (STUST) was placed behind the PET sheet.

Figure 7a–d showed the photographs of the PET sheet, pure UV coating deposited on PET sheet, UV coating/5 wt% PANDB and UV coating/10 wt% PANDB conductive composite films deposited on PET sheets, respectively. Figure 7c,d indicated that the UV coating/5 wt% PANDB and UV coating/10 wt% PANDB conductive composite films deposited on PET sheets both showed excellent translucency. The abbreviation of the school (STUST) was placed behind the PET sheet as the checking points for translucency.

**Figure 8:**
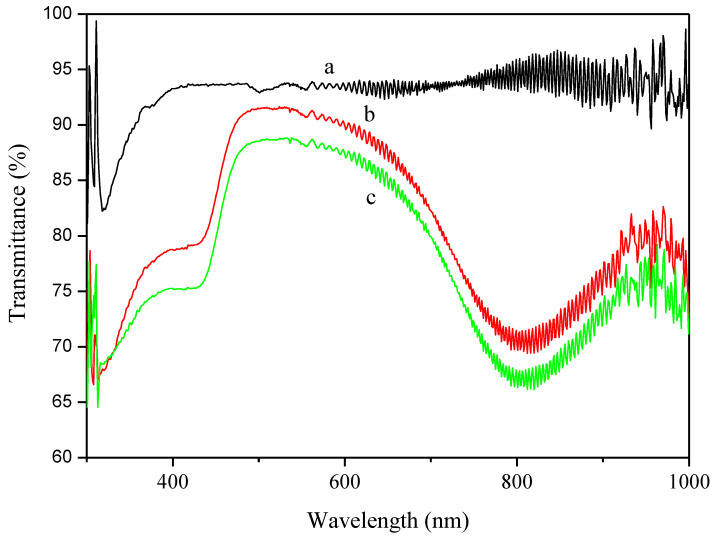
Transmittance spectra of (**a**) pure UV coating; (**b**) UV coating/5 wt% PANDB conductive composite film; and (**c**) UV coating/10 wt% PANDB conductive composite film deposited on PET sheets (using PET sheet as background).

Figure 8 showed the transmittance spectra of pure UV coating, UV coating/5 wt% PANDB conductive composite film, and UV coating/10 wt% PANDB conductive composite film deposited on PET sheets (using PET sheet as background). The translucency of these films at 550 nm showed a variation from 93.6% to 92.0% and 89.1% for the pure UV coating, UV coating/5 wt% PANDB conductive composite film, and UV coating/10 wt% PANDB conductive composite film deposited on PET sheets, respectively. From the experimental results, it was found that the more PANDB was added, the translucency of the conductive composite film was decreased. However, they all showed relatively good translucency as seen in Figure 7c,d. This was because the PANDB synthesized in xylene contained very good dispersion and solubility in xylene. Therefore, the UV coating/PANDB conductive composite film was very uniform and there was no obvious aggregation of PANDB in the UV curable coating. Moreover, from Figure 8b,c, the transmittance at the wavelengths of about 400 and 800 nm were lower than that of 550 nm, which indicated that UV coatings/PANDB conductive composite film could be used to block part of wavelength range. Due to this characteristic, this developed material might be able to be adopted in other applications.

**Figure 9:**
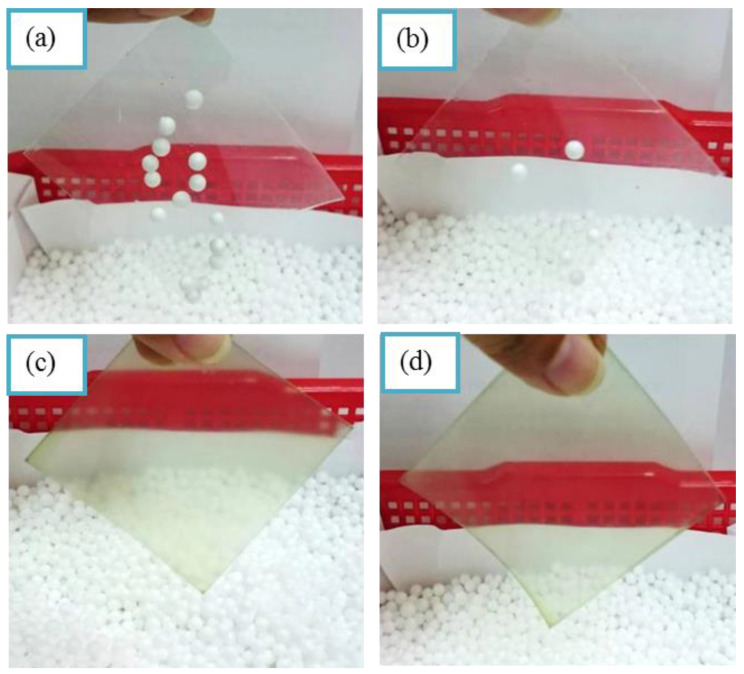
Photograph of antistatic comparison: (**a**) pure PET sheet; (**b**) pure UV coating film; (**c**) UV coating/5 wt% PANDB; and (**d**) UV coating/10 wt% PANDB conductive composite films.

The surface resistances of PET sheet, pure UV coating film, UV coating/5 wt% PANDB and UV coating/10 wt% PANDB conductive composite films were around 7.0 × 10^12^, 1.1 × 10^12^, 1.7 × 10^10^, and 1.5 × 10^9^ Ω/sq., respectively. Results implied the surface resistance could be decreased with increasing PANDB amount in UV coating. Figure 9 showed the antistatic comparison between the PET sheet, pure UV coating film, UV coating/5 wt% PANDB and UV coating/10 wt% PANDB conductive composite films. All test samples were abraded first and then placed into polystyrene (PS) foamed spheres. Obviously, the PS spheres were adsorbed on PET sheet and pure UV coating film as shown in Figure 9a,b. However, this observation was not found on the UV coating/5 wt% PANDB and UV coating/10 wt% PANDB conductive composite films as shown in Figure 9c,d. This phenomenon indicated that the UV coating/PANDB conductive composite film can be applied as antistatic products.

## 4. Conclusions

The conductive PANDB was synthesized directly in xylene by using a novel chemical oxidative polymerization with different reaction time at 25 °C. The results showed that most of the PANDB synthesized in xylene could be uniformly dispersed in xylene, and a small part of the PANDB could even be dissolved in xylene without forming any precipitation. On the contrary, for the PANDB synthesized in aqueous solution, it could not be uniformly dispersed in xylene, and part of PANDB could even be precipitated in xylene. The influences of synthesis time on properties of PANDB synthesized in xylene were also examined. The results illustrated that if the reaction time was too short, the chemical oxidation reaction was insufficient, and if the reaction time was too long, it resulted in an excessive oxidation and other side reactions. Both phenomena can affect the conductivity of the product. In this synthesis system, the optimum reaction time was found and then set as 24 h. Its conductivity at this optimized condition was around 2.03 S/cm. FE-SEM images showed that different synthesis conditions resulted in different surface morphology of PANDB. The more needle-like particles founded in the synthesized PANDB, the greater conductivity the product will have. The synthesized PANDB was blended with UV curable coating firstly and then coated on PET sheet. The UV coating/5 wt% PANDB and UV coating/10 wt% PANDB conductive composite films displayed an impressive translucency (around 92.0 and 89.1% at 550 nm) along with an adequate flexibility at room temperature. Furthermore, the functional UV coating/PANDB conductive composite film can be applied as antistatic products and biosensors.
